# Supplementary material for: Development, validation, and reliability testing of the College Perspectives around Food Insecurity survey
Source: PLoS One. 2025 Jan 28;20(1):e0317444. doi: 10.1371/journal.pone.0317444 (PMC11774399; doi:10.1371/journal.pone.0317444)
Supplement: S1 Table — (PDF) [file pone.0317444.s001.pdf]

S1 Table

Question (Theoretical Concept)	Branching Questions (Theoretical Concept)
**Students' Experiences with Food Section**	
Q1: I prepare food and/or cook meals. (B)[1]	If Q1=yes, then:
	• Q2: I have enough space to prepare food and/or cook meals. (E)[2]
	• Q3: The space I have to prepare food and/or cook meals is unclean or untidy (such as dirty dishes or dirty surfaces). (E)[2]
	• Q4: I do not have access to cooking equipment (such as pans, mixing bowls, utensils, etc.). (E)[2]
	If Q4=agree or strongly agree, then:
	○ Q5: Not having access to cooking equipment (such as pans, mixing bowls, utensils, etc.) makes it difficult to prepare and/or cook food for myself. (E)[2]
	• Q6: I do not have access to kitchen appliances (such as a refrigerator, freezer, stove, oven, microwave, etc.). (E)[2]
	If Q6=agree or strongly agree, then:
	○ Q7: Not having access to kitchen appliances (such as a refrigerator, freezer, stove, oven, microwave, etc.) makes it difficult to prepare and/or cook food for myself. (E)[2]
	• Q8: I rarely have time to prepare food and/or cook meals for myself. (B)[2]
	• Q9: To save money, I prefer to prepare food and/or cook meals for myself. (P)[2]
Q10: I am confident in my ability to prepare food and/or cook meals. (P)[2]	--
Q11: I have enough space to store food. (E)[2]	If Q11=strongly disagree, disagree or neither agree nor disagree, then:
	• Q12: I buy smaller packages of food because I have a limited amount of storage space. (B)[2]
**Shopping and Obtaining Food Section**	

Q13: In which of these ways do you obtain food? Mark all that apply. (B)[3]	If Q13= buying food at a grocery store, convenience store, farmers market or through community supported agriculture (CSA), then:
	• Q14: I rely on foods sold at discounted prices as a strategy to help me save money. (B)[2]
	• Q15: I prioritize spending money on entertainment activities over buying food. (P)[2]
	• Q16: I prioritize other living expenses (anything you pay for other than food) over buying food. (P)[2]
	If Q13=receiving money from family to buy food, then:
	• Q17: I am only able to pay for food with help from my family (E)[2]
	If Q13=receiving money from friends to buy food, then:
	• Q18: I am only able to pay for food with help from my friends. (E)[2]
	If Q13=purchasing a campus meal plan/utilizing dining dollars, then:
	• Q19: I do not have enough money to buy the campus meal plan I want. (P)[2]
	• Q20: I purchase the least expensive campus meal plan as a strategy to help me save money (B)[2]
	• Q21: I worry about getting enough to eat through my current campus meal plan (P)[2]
	• Q22: Having a campus meal plan is stressful to me. (B)[2]
	• Q23: Have you ever had meals left over on your campus meal plan at the end of the semester/term/quarter? (B)[4]
	If Q23=yes, then:
	○ Q24: I have had meals left on my campus meal plan because the dining hall is not open at convenient times for me. (E)[2]
	○ Q25: I have had meals left on my campus meal plan because I have other commitment(s) (jobs, sports practice, club meeting, etc.) that conflict with dining hall hours. (E)[2]
	○ Q26: I have had meals left on my campus meal plan because I don't want to go to the dining hall alone. (P)[2]

	○ Q27: I have had meals left on my campus meal plan because my friend uses their campus meal plan for my meals (swipes me into the dining hall). (B)[2]
	If Q13=buying food from a fast-food or sit-down restaurant:
	• Q28: I prioritize other living expenses (anything you pay for other than food) over eating out at fast-food or sit-down restaurants. (P)[2]
	• Q29: I skip meals so I have enough money to eat out with friends at fast-food or sit-down restaurants. (B)[2]
	• Q30: I use coupons/discounts to save money when eating out at fast-food or sit-down restaurants. (B)[2]
	• Q31: On average, I spend more money in a month on eating out at fast-food and sit-down restaurants than buying groceries. (B)[2]
	• Q32: I spend money eating out at fast-food or sit-down restaurants because I feel pressure from my friends and/or others to do so. (B)[2]
	If Q13=receiving free meals or discounted meals through your job/work, then
	• Q33: I would not have enough food to eat without free meals or food discounts from my job/work. (E)[2]
	• Q34: I seek out free or discounted food at my job/work to save money. (B)[2]
	• Q35: How often do you receive free/discounted food from your job/work? (E)[5]
Q36: I spend money on entertainment activities (does not include eating out) because I feel pressure from friends and/or others to do so (B)[2]	--
Q37: I am currently unable to follow my cultural eating patterns* because it costs too much money to buy my cultural foods. (P)[2]	--
Q38: I am currently unable to follow my cultural eating patterns* because my cultural foods are not available where I shop. (P)[2]	--

Q39: I worry about getting enough to eat with the current amount of money I have to spend on food. (P)[2]	--
Q40: I accept free food on campus as a way to help me save money. (B)[2]	--
Q41: I share food costs (groceries, fast food, sit-down restaurant, etc.) with someone else as a strategy to help me save money. (B)[2]	--
Q42: It costs too much money for me to eat healthy. (P)[2]	--
Q43: I skip meals because I do not have enough time. (B)[2]	
Q44: Skipping meals is a strategy I use to help me save money. (B)[2]	
**Food Assistance Program Experience Section**	
Q45: Which of these food assistance programs did your family ever use when you were growing up (before you started college)? Mark all that apply. (E)[6]	--
Q46: The following statement will address your <u>current</u> experience with food assistance programs. Food stamps or SNAP (Supplemental Nutrition Assistance Program) (E)[7]	If Q46=I know or have heard about this food assistance program but do not currently use it, then:
	• Q47: I do not know how to apply for this food assistance program (P)<br>• Q48: I previously applied for this food assistance program but did not qualify (P)<br>• Q49: I have not applied for this food assistance program but I do not believe I would qualify (E)<br>• Q50: I do not need this food assistance program (P)
Q51: WIC (Special Supplemental Nutrition Program for Women, Infants and Children) (E)[7]	If Q51=I know or have heard about this food assistance program but do not currently use it, then:
	• Q52: I do not know how to apply for this food assistance program (P)

	• Q53: I previously applied for this food assistance program but did not qualify (P)<br>• Q54: I have not applied for this food assistance program but I do not believe I would qualify (E)<br>• Q55: I do not need this food assistance program (P)
Q56: Campus food pantry/food bank (E)[7]	--
Q57: Community food pantry/food bank (E)[7]	--
Q58: Soup kitchen (E)[7]	--
Q59: Resources from a church of faith-based group (E)[7]	--
	If Q46, Q51, Q56, Q57, Q58 or Q59=I currently use this food assistance program, then:
	Q60: I eat healthier because I use a food assistance program. (E)[2]
	Q61: Food assistance programs allow me to have enough food during the month. (E)[2]
	Q62: I feel that there is a stigma attached to those using food assistance programs. (P)[2]
**Transportation Methods Section**	
Q63: What types of transportation do you use to get food? Mark all that apply.[9]	If Q63=own motorcycle or moped, bicycle, bus/train, and/or walk, then:
	• Q64: The fact that I must transport food back with me limits the amount I get (E)[2]
	If Q63=borrow someone else's car, motorcycle, moped, and/or bicycle, then:
	• Q65: I feel uncomfortable asking to borrow someone else's car/bicycle/moped/motorcycle to get food. (P)[2]
	If Q63=ask someone else for a ride, then:
	• Q66: I feel uncomfortable asking someone else for a ride to get food. (P)[2]
Q67: I feel I have to travel too far from where I live to access healthy food (P)[2]	--
**Alcohol Consumption Section**	

Q68: Do you drink alcohol? (B)[1]	If Q68=yes, then:
	• Q69: How many drinks do you have a week? (One drink is defined as 12 ounces of beer, 5 ounces of wine, 1.5 ounces of 80-proof distilled spirits.) (B)[10]
	• Q70: How often are you drinking in a week? (B)[11]
	• Q71: I don't have enough money to buy food because I spend too much money on alcoholic beverages. (P)[12]
**Managing Finances Section**	
Q72: How are you paying for your college expenses (tuition, books, rent, etc.)? Mark all that apply. (E)[13]	--
Q73: I worry about having enough money to pay for college expenses (books, tuition, rent, etc.). (P)[2]	--
Q74: Saving money is important to me. (P)[2]	--
Q75: I am able to save money. (P)[2]	If Q75= agree or strongly agree, then:
	• Q76: Having money saved helps me have peace of mind. (P)[2]
Q77: Have you ever run out of money? (B)[1]	If Q77=yes, then:
	• Q78: What do you rely on when you run out of money? (B)[14]
	○ If Q78= credit card, then:
	○ Q79: I pay off my credit card bill in full every month. (B)[2]
	If Q79=strongly disagree, disagree, or neither agree nor disagree, then:
	▪ Q80: I have accrued/incur credit card debt to buy food. (B)[2]
	▪ Q81: I am concerned about my outstanding credit card debt. (P)[2]
	• Q82: When I run out of money, I stretch the food I have to last until I have more money. (B)[2]
**Campus Strategies Section**	
Q83: Universities should discount campus meal plans for students who need it. (P)[2]	--

Q84: Students with campus meal plans should be able to donate extra meals to students in need. (P)[2]	--
Q85: Extra food from food establishments on campus should be made available to students who need it. (P)[2]	--
Q86: Food options on campus should be less expensive. (P)[2]	--
Q87: Universities should provide a grocery store on campus with discounted food prices. (P)[2]	--
Q88: Universities should require all students to learn about financial management and budgeting. (P)[2]	--
Q89: Universities should require all students to learn about menu planning. (P) [2]	--
Q90: Universities should require all students to learn about strategies to save money when shopping. (P)[2]	--
Q91: Universities should require all students to learn about food preparation and/or cooking meals. (P)[2]	--
Q92: Universities should pay student employees a higher hourly wage. (P)[2]	--
Q93: Universities should increase access and/or promotion of a campus food pantry. (P)[2]	--
Q94: Universities should increase access and/or promotion of free food events on campus. (P)[2]	--
Q95: Universities should increase access and/or promotion of food stamps or SNAP (Supplemental Nutrition Assistance Program) for eligible students. (P)[2]	--
Q96: Universities should increase access and/or promotion of WIC (Special Supplemental Nutrition Program for Women, Infants and Children) for eligible students. (P)[2]	--
**COVID-19 Section**	
Q97: At any time during the COVID-19 pandemic, I was not able to get enough food to eat. (E)[2]	--

Q98: At any time during the COVID-19 pandemic, I could not afford to eat balanced meals. (E)[2]	--
Q99: At any time during the COVID-19 pandemic, I worried whether my food would run out before I got money to buy more. (P)[2]	--

E=Environment, B=Behavior, P=Personal

*Defined on survey as “preferred foods that you grew up eating or that are familiar to you.”

[1]Response options: yes, no

[2]Response options: strongly disagree, disagree, neither agree nor disagree, agree, strongly agree

[3]Response options: buying food at a grocery store or convenience store, buying food at a farmers market or through community supported agriculture (CSA), purchasing a campus meal plan/utilizing dining dollars, buying food from a restaurant/fast food, buying food from a vending machine, receiving free meals or discounted meals through job/work, receiving money from family to buy food, receiving money from friends to buy food, through food assistance programs (SNAP, WIC, food bank/pantry, etc.), through free food events on campus, other

[4]Response options: yes, no, I have not finished a semester/term/quarter before

[5]Response options: Less than 1 time per week, 1-2 times per week, 3-4 times per week, 5-6 times per week, 7+ times per week

[6]Response options: Food stamps or SNAP (Supplemental Nutrition Assistance Program), WIC (Special Supplemental Nutrition Program for Women, Infants, and Children), free or reduced price school meals, community food pantry/food bank, soup kitchen, resources from a church or a faith-based group, other, I don’t know, my family did not use any of these food assistance programs

[7]Response options: I currently use this food assistance program, I used this food assistance program in the past but do not currently use it, I know or have heard about this food assistance program but do not currently use it, I do not know or have not heard about this food assistance program and I do not currently use it

[8]Response options: I do not know how to apply for this food assistance program, I previously applied for this food assistance program but did not qualify, I have not applied for this food assistance program but I do not believe I would qualify, I do not need this food assistance program

[9]Response options: I use my own car, I use my own motorcycle or moped, I use my own bicycle, I borrow someone else's car, I borrow someone else's motorcycle or moped, I borrow someone else's bicycle, I take taxi/Uber/Lyft, I ask someone else for a ride, I take the bus/train, I walk, I order food or groceries online and have it delivered, other

[10]Response options: less than 1 drink, 1-2 drinks, 3-4 drinks, 5-6 drinks, 7-8 drinks, 9 or more drinks

[11]Response options: less than 1 time per week, 1-2 times per week, 3-4 times per week, 5-6 times per week, 7-8 times per week, 9 or more times per week

[12]Response options: never, rarely, sometimes, very often, always

[13]Response options: parents or other family members' financial support, spouse, friends' financial support, sponsoring company/organization or person, student loans, grants, scholarships, own job/employment, own savings/investments, own credit card, other
[14]Response options: credit card, other people, other (please specify)
