# Supplementary material for: Development, validation, and reliability testing of the College Perspectives around Food Insecurity survey
Source: PLoS One. 2025 Jan 28;20(1):e0317444. doi: 10.1371/journal.pone.0317444 (PMC11774399; doi:10.1371/journal.pone.0317444)
Supplement: Attachment — Submitted filename: Response to Reviewers.docx [file pone.0317444.s002.docx]

December 4, 2024

Dear Academic Editor and Reviewers,

We appreciate the time you spent reviewing our manuscript and providing us with feedback. Please find our responses to your feedback in the table below.

PONE-D-24-06404

Responses to Reviewers

| **Journal/Reviewer Comments** | **Authors’ Responses** |
| --- | --- |
| ***Journal Requirements*** |  |
| Please ensure that your manuscript meets PLOS ONE's style requirements, including those for file naming. The PLOS ONE style templates can be found at  https://journals.plos.org/plosone/s/file?id=wjVg/PLOSOne_formatting_sample_main_body.pdf and  https://journals.plos.org/plosone/s/file?id=ba62/PLOSOne_formatting_sample_title_authors_affiliations.pdf | We revised our manuscript based on PLOS ONE’s style requirements.  We also ensured that our file names matched the style requirements. |
| Thank you for stating the following financial disclosure:  Internal funding from Brigham Young University.  Please state what role the funders took in the study.  If the funders had no role, please state: "The funders had no role in study design, data collection and analysis, decision to publish, or preparation of the manuscript."  If this statement is not correct you must amend it as needed.  Please include this amended Role of Funder statement in your cover letter; we will change the online submission form on your behalf. | The funders had no role in the study, so we included that statement in our cover letter. Thank you for making this correction on our behalf. |
| In the online submission form, you indicated that your data is available only on request from a third party. Please note that your Data Availability Statement is currently missing the name of the third party contact or institution / contact details for the third party, such as an email address or a link to where data requests can be made. Please update your statement with the missing information. | We modified this statement to include the contact information of the third party. In this case, the data sharing agreement form gets first approved through Brigham Young University’s IRB administrator. We added the office’s email address as a contact point for establishing the data sharing agreement.  In the online submission form, it now states:  Data cannot be shared publicly because a data sharing agreement would need to be put in place first to stipulate how the data can be used. Data are available for institutions willing to complete a data sharing agreement with Brigham Young University ([irb@byu.edu](mailto:irb@byu.edu)) that gets approved through the researcher's institutions. |
| ***Reviewer #1:*** |  |
| The study aims to understand the impact of food insecruity in college student population. The authors tested the survey reliability.  I think the authors should include a discussion about survey participant recruitment as well as how they will reduce biases during recruitment of certain individuals wanting to participate in surveys. They short of touch the subject. I think that is an important component of the survey study. | Thanks for this feedback.  We added content to the participant recruitment section to clarify our recruitment approach. See lines 104-121.  We added additional text in the limitations section to acknowledge the possibility of bias being introduced into our study based on using non-probability sampling strategies. See lines 403-405. |
| ***Reviewer #2:*** |  |
| The manuscript by Jennette Kilgrow et al discusses the development, validation, and reliability testing of a survey on college perspectives around food insecurity. The main goal of this study was to develop and test the validity and reliability of a survey aimed at evaluating internal and external factors related to college food insecurity. The authors have used a mixed methods approach to evaluate the College Perspectives on Food Insecurity survey. However, the manuscript could proceed further after the modification following the comments below. | We appreciate the reviewer’s time in evaluating our manuscript and for the feedback provided to us. |
| The introduction of this manuscript is poorly written. It should be modified with more relevant references. | We revised the Introduction for clarity. We also added updated citations to more recent publications on food insecurity and college students. Please see lines 53-82. |
| The results of this survey are necessary to show a graph including the participants' details. | Researchers provide demographic information about students who participated in cognitive interviews (phase 3) and the pilot study (phase 4). Please see Tables 1 and 2.  Because the intent of this study was to evaluate validity and reliability of survey questions, researchers only presented data relevant to these aims. Thus, the present study provides details about how researchers developed the survey based on feedback from expert reviewers and students. Students’ responses to the various survey questions will be presented in future manuscripts with a larger (and different) sample from the present study. |
| The references are not updated. | We appreciate this feedback. We updated the references in the Introduction to those published within the past 5 years.  We also updated the references in the Discussion to those published in the past 5 years when more recent, relevant works were available.  We did not update the references cited in the Methods section because those were the works that we used throughout the research study period. |
| There is no statistical analysis. In my point of view, a statistical analysis could help to understand the results of the survey. | Researchers added chi-square statistics and Fisher’s exact test (when > 20% of expected cell counts were <5) to compare differences between students with food security vs. food insecurity for the reliability testing phase 4. A p-value <0.05 was considered significant. Please see lines 245-248 and Table 2.  Given the small sample sizes in the cognitive interviews phase 3, no statistical comparisons were calculated. We used descriptive statistics to display the demographics data in Table 1. However, we noted that this was not described in the Analysis section. We have added this text in lines 232-234.  Researchers also used descriptive statistics (means) to evaluate expert reviewers’ feedback on each survey question related to clear phrasing, importance, and content appropriateness. A threshold of 8.0 (on average) was considered for evaluating content validity. This is described in lines 221-224.  Researchers evaluated survey reliability through test-retest statistics and Cronbach-alpha statistics. This is described in lines 236-241 (note that some modifications in text were made for clarity).  As mentioned previously, the intent of this study was to present the survey development process used to evaluate survey validity and reliability. Students’ responses to the various survey questions will be presented in future manuscripts with a larger (and different) sample from the present study. |
